# Investigation of cypermethrin toxicity in Swiss albino mice with physiological, genetic and biochemical approaches

**DOI:** 10.1038/s41598-022-15800-8

**Published:** 2022-07-06

**Authors:** Baran Seven, Kültiğin, Çavuşoğlu, Emine Yalçin, Ali Acar

**Affiliations:** 1 Department of Biology, Institute of Science, Giresun University, Giresun, Turkey; 2 Department of Biology, Faculty of Science and Art, Giresun University, Giresun, Turkey; 3 Department of Medical Services and Techniques, Vocational School of Health Services, Giresun University, Giresun, Turkey

**Keywords:** Cell biology, Genetics

## Abstract

In this study, cypermethrin toxicity was investigated using physiological, biochemical and cytogenetic parameters, and more than one organ and cell type was preferred to determine these effects. In this multifaceted study, the genotoxicity mechanism of cypermethrin was elucidated by molecular docking. In addition, comet assay technique was applied to detect and quantify DNA damage at the cell level. For this aim, body and organ weights, aspartate aminotransferase (AST), alanine aminotransferase (ALT), malondialdehyde (MDA), glutathione (GSH), blood urea nitrogen (BUN) and creatinine levels, mitotic index (MI), DNA fragmentation, frequency of micronucleus (MN) and chromosomal aberrations (CAs) were used as indicators of toxicity. Mice were divided into 4 groups. The control group was fed with tap water and the administration groups were *orally* exposed to 62.5, 125 and 250 mg/kg b.w cypermethrin for 28 days. Then, the mice were sacrificed and tissue samples were collected. Cypermethrin caused a decrease in body and organ weights, GSH levels and MI and an increase in AST, ALT, MDA, BUN, creatinine levels and the frequency of MN and CAs (break, ring, gap, acentric, etc.). Cypermethrin promoted MN formation in leukocyte, erythrocyte, buccal mucosa epithelial cells. CAs and MN formation promoted by cypermethrin have been associated with DNA-cypermethrin interactions. This interaction has been demonstrated by simulation with molecular docking method and experimentally by spectral measurements of DNA. As a result, all three doses of cypermethrin caused toxicity in different cell types. In other words, the effect of cypermethrin taken into the body was not limited to only one cell type or region. Therefore, cypermethrin is a pyrethroid insecticide that promotes multifaceted toxicity in non-target organisms.

## Introduction

Pesticides are defined as any chemical substance designed to destroy, prevent or control any pest and unwanted plant or animal species. In other words, they are chemicals used to prevent insect, spider or other pest damage during the production, processing, storage and marketing of agricultural products. Pesticides are toxic chemicals that are deliberately released into the environment. Although pesticides are classified in many different ways, the most widely used classification is based on the type of target organism. According to the classification made in this way, pesticides are classified as herbicides, fungicides, rodenticides, acaricides and insecticides^1^. Pesticide production and use are increasing day by day for reasons such as providing high quality insect-free crop production, preventing human diseases transmitted by vectors, being very economical for farmers and increasing the amount of food production. Despite all these benefits, pesticides are highly toxic chemicals for both humans and the environment. They are highly toxic due to their environmental resistance and bioaccumulation properties. They can cause environmental pollution as well as pesticide residues in agricultural products and foodstuffs. By entering the atmosphere, pesticides can be transported long distances or reach non-target organisms by contact, dermal, oral and respiratory routes. It is known that only 1% of the total amount of pesticides applied for weed and pest control can reach the targeted pests. The rest may cause undesirable effects on the environment and non-target organisms including humans. When pesticides reach non-target organisms, they undergo biotransformation through reactions such as oxidation, hydrolysis, conjugation or reduction and can eventually produce more toxic metabolites.

Pesticides such as insecticides can induce acute damage to organisms even at low concentrations, while long-term exposure can cause genetic disorders and physiological changes that shorten lifespan^1–3^. Previous studies on insecticides have reported that these chemicals cause oxidative stress, liver and kidney damages in experimental animals. In addition, occupational exposure to insecticides accounts for approximately 4% of all human cancers^4,5^.

One of the widely used insecticides in the world is cypermethrin. Cypermethrin is a fourth-generation synthetic pyrethroid group broad-spectrum insecticide used for pest control and crop loss prevention in agriculture. Its widespread use can cause various toxic effects on non-target organisms. Cypermethrin is a highly toxic chemical that can be inhaled, ingested and dermally. Exposure can cause skin irritation, numbness and tingling, itching and burning sensation in the eyes, loss of bladder control, seizures and after a while, death. Due to its lipophilic structure, it has been found to accumulate in body fat, skin, liver, kidneys, adrenal glands, ovaries and brain. The nervous and muscular systems are the main body parts affected. Toxic oral doses in mammals are 100–1000 mg/kg and potentially lethal acute oral doses are 10–100 g. The short-term neurotoxicity caused by cypermethrin occurs through hyper-stimulation of the central nervous system. Cypermethrin can cause neurotoxicity by modulating gamma-aminobutyric acid (GABA) levels or by promoting the formation of free radicals^6–8^.

In this study, the potential toxic effects of cypermethrin on albino mice were investigated with a multidisciplinary approach by using biochemical, physiological and cytogenetic parameters. While physiological effects were determined by examining the feed consumption, liver, kidney and body weights, biochemical effects were investigated by alterations in oxidant/antioxidant dynamics and serum parameters. Chromosomal aberrations (CAs) and mitotic index (MI) in bone marrow cells, micronucleus (MN) frequency in leukocytes, erythrocytes and buccal mucosa epithelial cells were investigated for cytotoxic and genotoxic effects. DNA fragmentation caused by cypermethrin was investigated by Comet test and the effects of cypermethrin on cell viability were tested by trypan blue test. The toxicity mechanism was evaluated by associating each parameter with each other. In order to elucidate the mechanism of genotoxic effects, cypermethrin-DNA interaction was tested by molecular docking and spectral measurement methods.

## Material and methods

### Test material and animal care

24 healthy male *Mus musculus* var. *albinos* were used as the test material, and cypermethrin (CAS number 52315-07-8), a Sigma-Aldrich product, was used as the test chemical. During the experiment, albino mice were kept in 26 × 15 × 50 stainless steel cages, at 22 ± 3 °C and 55 ± 5% relative humidity, on a 12-h light/12-h dark cycle basis. All experiments were performed in accordance with the guidelines of the Animal Experiments Local Ethics Committee of Giresun University and approved by the Animal Ethics Committee of Giresun University (protocol number: 2010/01). This study was carried out in compliance with the ARRIVE guidelines.

### Experiment protocol

Swiss albino mice were randomly divided into 4 groups, with 6 mice in each group.

- Group I: tap water + standard pellet feed (control group).
- Group II: 62.5 mg/kg b.w cypermethrin + standard pellet feed.
- Group III: 125 mg/kg b.w cypermethrin + standard pellet feed.
- Group IV: 250 mg/kg b.w cypermethrin + standard pellet feed.

**Figure 1:**
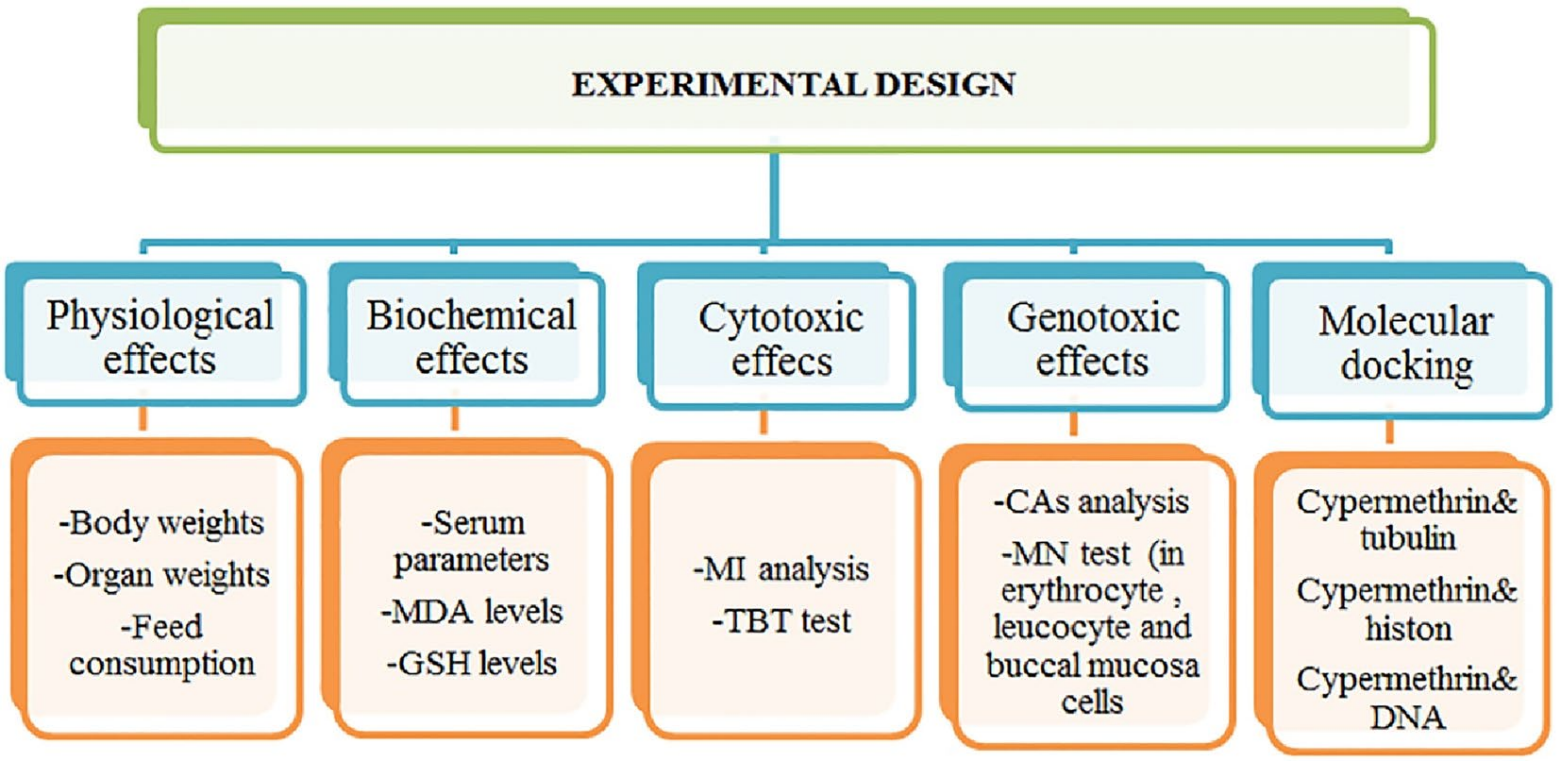
Experimental design.

For 28 consecutive days (4 weeks), the mice in the control group were fed with tap water, and the mice in the treatment group were fed orally with 62.5, 125 and 250 mg/kg b.w of cypermethrin. In a limited number of studies investigating the toxic effects of cypermethrin in mice, the 50–250 mg/kg dose range of cypermethrin was preferred. In this study, 62.5, 125 and 250 mg/kg doses of cypermethrin were preferred as multiples of each other in accordance with the dose ranges in the literature. The water, feed and cypermethrin solution were checked daily. In order to adapt to the environmental conditions, the mice were brought to the laboratory where the experiment would be conducted 7 days before the application period. At the end of the 28-day administration period, all mice were sacrificed. During the experiment, clinical symptoms of all animals such as activity, irritability, diarrhoea, gross tremor, limb paralysis, wounds and mortality were monitored daily. Feed and water intakes were also noted. All investigated parameters were summarized in Fig. 1.

### Weight measurements

After the Swiss albino mice were stunned by administering halothane anesthetic (0.05%), their final body weights were measured at the beginning of the first day of the administration period and at the end of the 28-day consecutive administration period with the help of precision balances. In addition, after sacrificing the mice at the end of the 28th day, liver and kidney organ weights were also measured with the help of sensitive balance.

### Feed consumption

Considering that each albino mouse consumes approximately 3–4 g of feed per day^9^, 5 g (*above the daily consumption amount*) of feed for each mouse in each group, 30 g for a total of 6 mice, and 210 g per week was put into the feeders in the cages. During the 28-day (*4-week*) application period, the feed consumption was calculated by measuring the remaining feed amount in the container at the end of the 7th day of each week.

### Serum analysis

To obtain serum from whole blood, mice were knocked unconscious under Halothane anesthesia (0.05%), and then blood samples were taken into vacutainer tubes, centrifuged at 1.200 g for 10 min. at + 4 °C in a cold environment, and stored at − 20 °C until analysis. AST (AST/GOT Liquid Reagent, CAT. NO: A559-150) and ALT (ALT/GPT Liquid Reagent, CAT. NO: A524-150) enzyme activities, BUN (CAT. NO: B549-150) and creatinine (CAT. NO: C513-480) levels were measured with the help of Medispec 99 M autoanalyzer using commercially available kits (Teco Diagnostics, USA)^10,11^.

### Effects on antioxidant/oxidant dynamics

In order to determine the effects of cypermethrin on antioxidant/oxidant balance MDA and GSH levels were measured in kidney and liver. For this aim, each mouse in the experimental groups was sacrificed by heart exsanguination method under Halothane anesthesia (0.05%), liver and kidneys were removed, washed, dried in a sterile environment and prepared for biochemical analysis. Liver and kidney tissues were homogenized (homogenizer, Ultraturrax Type T25-B, IKA Labortechnik, Germany) in an ice-cold 0.15 M KCl bath at 16,000 rpm for 3 min., the homogenates were centrifuged at 5000*g* at 4 °C for 1 h, and the supernatants were taken and stored at − 40 °C until analysis^12^. Tissue MDA and GSH levels were measured according to the colorimetric method suggested by Yoshioka et al.^13^ and Beutler et al.^14^ in the UVmini-1240 (Shimadzu, Japan) brand UV-spectrophotometer.

### Cytotoxic and genotoxic effects

MI and TBT tests were used to evaluate the cytotoxic effects of cypermethrin, and MN and CAs analyses were used to determine the genotoxic effects. MI and CAs tests were performed on bone marrow cells, MN test on buccal mucosal epithelial, erythrocyte and leukocyte cells, and TBT test on liver and kidney cells.

### Trypan blue test

The trypan blue test (TBT) allows the determination of the amount of live and dead cells (cell viability) using trypan blue, a negatively charged dye. TBT was carried out according to the method suggested by Strober^15^. Before use, one part of 0.4% trypan blue which was filtered through a sterile filter, and one part of cell suspension (separately for liver and kidney), were mixed in a tube, the mixture was placed on slides and incubated for 3 min. at room temperature. After mixing with trypan blue, the cells were examined under a light microscope within 3–5 min. in order not to cause cell death and decrease in the number of viable cells, and counting processes were carried out. A total of 600 cells were counted, 100 for each mouse in each group.

### Buccal mucosal epithelium MN test

In order to detect the presence of MN in the buccal mucosal epithelial cells, each mouse in the experimental groups was stunned with the help of Halothane anesthesia (0.05%), the mouths of the mice were rinsed with distilled water, and the mucous epithelial cells were obtained by scanning the right and left buccal mucosa with a slightly moist and blunt toothpick without too much pressure. Cells were placed on sterile slides and allowed to dry for 15 min. At the end of the time, cells were fixed in a solution of 3 volumes of methanol and 1 volume of acetic acid for 10 min., stained with Feulgen and Fast Green, and coverslip covered with Entellan^16^. 1000 cells were analyzed in each group for MN frequency in buccal mucosal epithelial cells.

### Erythrocyte MN Test

Mouse Erythrocyte MN assay was performed according to the method suggested by Te-Hsiu et al.^17^. Mice in the experimental groups were stunned with Halotene anesthetic (0.05%), blood samples were collected from their tail veins with a small needle syringe, approximately 5 µL of the collected blood was mixed with 3% EDTA solution and spread on sterile slides. The samples on the slide were fixed in 70% ethanol for 2 min. and left to dry at 21 °C for 24 h. At the end of the time, the slides were stained with 5% Giemsa for 15 min. and covered with a coverslip with entellan. 1000 cells were analyzed in each group for MN frequency in erythrocyte cells.

### Leukocyte MN Test

For the leukocyte MN test, blood samples from each mouse were centrifuged at 5000 rpm for 10 min. and the upper clear part was removed. 5 mL of 0.075 M KCl solution was added to the remaining residue and left at room temperature for 20 min. At the end of the time, the tubes were centrifuged again at 5000 rpm for 10 min. Again, the upper part was removed. 5 mL of a washing solution consisting of 3 volumes of methanol and 1 volume of glacial acetic acid was added to the remaining residue and kept at − 20 °C for 30 min. At the end of the period, leukocyte cells were spread on sterile slides, stained with 5% Giemsa and covered with a coverslip with Entellan^18^. 1000 cells were analyzed in each group for MN frequency in leukocyte cells.

The criteria proposed by Fenech et al.^19^ were taken into account to determine MN formation in buccal mucosal epithelium, erythrocyte and leukocyte cells. According to this:

- The dimensions of the MN should be approximately 1/3 of the cell nucleus,
- When MN is stained, it should take on the color that the cell nucleus was stained,
- Even when the membranes of the MN and cell nuclei come into contact with each other, the boundary between them must be clearly defined.

In all three cell types, 1000 cells in each group were counted to detect the presence of MN and photographed at 500× magnification under the Irmeco IM-450 TI model research microscope.

### Chromosomal analysis and mitotic index

Mice given 0.025% colchicine intraperitoneally 2 h before sacrification were sacrificed under Halotene anesthesia (0.05%) at the end of the period. Bone marrow was aspirated from the femurs of mice. It was washed with physiological saline and treated with 0.075 M KCl. Afterwards, it was fixed with Carnoy’s and stained with 5% giemsa^20^. CAS were detected in the IM-450 TI model research microscope and classified according to the criteria reported by Savage^21^. In prepared slides, MI was determined as the percentage of dividing cells among 1000 nucleated cells in slides prepared for each group.

### Comet assay (single-cell gel electrophoresis)

The protocol of Tice et al.^22^ was performed for alkaline single cell gel electrophoresis with slight modifications. Slides were dipped in 1% normal melting point agarose for coating and allowed to dry at 37 °C. 10 µL of peripheral blood were added to 120 µL of 0.5% low-melting point agarose at 37 °C, layered onto a coated slide, covered with a coverslip and left at 4 °C for 5 min. to solidify the agarose. The coverslip was removed and the slides were immersed into a lysis solution for approximately 1 h. After lysis, the slides were transferred to a horizontal gel electrophoresis tank with a fresh and cooled alkaline buffer. After a 20-min. unwinding period, the DNAs of the groups were electrophoresed at 0.86 V/cm (20 V, 300 mA) for 20 min. Slides were stained using ethidium bromide solution after carefully flushing three times with tris-buffer (0.4 M tris, pH 7.5) for 5 min. The preparations were washed with cold water to remove excess stain and covered with a coverslip. To prevent DNA damage, all steps were performed in low light and analyzed by fluorescence microscopy. Cells appearing as comets were evaluated by Comet Assay software version 1.2.3b^23^ with the parameters of tail DNA percentage, tail moment and olive tail moment and 600 cells were calculated for each group.

### Molecular docking

Molecular docking was performed to analyze potential interactions of cypermethrin with DNA molecules. The 3D structures of B-DNA dodecamer (PDB ID: 1bna)^24^, B-DNA dodecamer d (PDB ID: 195d)^25^ and DNA (PDB ID: 1cp8)^26^ molecules were obtained from the protein data bank. The 3D structure of cypermethrin (PubChem CID: 2912) was retrieved from PubChem. It was prepared for molecular docking by determining the active sites of DNA molecules, removing water molecules and ligands, and adding polar hydrogen atoms. Energy minimization of the 3D structure of cypermethrin was accomplished with the uff-force field employing Open Babel v.2.4.0 software^27^. The receptor molecules were allocated Kollman charges, whereas cypermethrin was assigned Gasteiger charges. The molecular docking process was carried out with the grid box containing the entire structure of DNA molecules. Then docking was performed using Autodock 4.2.6 software^28^ based on Lamarckian genetic algorithm. The docking analysis and 3D visualizations were performed with Biovia Discovery Studio 2020 Client.

### DNA extraction and spectral measurements

Spectral measurements were performed to confirm the DNA-cypermethrin interactions. For this purpose, DNA was first obtained from blood of mice. The cetyltrimethyl ammonium bromide (CTAB) method recommended by Miladinov^29^ was used for DNA extraction from blood. Whole blood samples were taken from the tail veins of the mice with the help of a fine-tipped syringe. The samples were transferred to 2 mL tubes containing EDTA. 500 µL of solution I (8% CTAB, 1.5 M NaCl, 100 mM TRIS pH 8.5, 50 mM EDTA pH 8) pre-warmed at 68 °C was mixed with 250 µL of blood sample and incubated at 68 °C for 30 min. Then, 750 µL of chloroform was added and mixed by inverting several times then centrifuged for 5 min. at 13,000 rpm at 21 °C. The volumes were determined by transferring the DNA containing the upper phase and the proteins containing the middle phase to new tubes without deterioration. The aqueous phases were mixed with solution II (5% CTAB, 0.1 M NaCl) and dH_2_O and centrifuged at 13,000 rpm for 5 min. at 21 °C. Supernatants were discarded and pellets were re-suspended in 250 µL of 1.2 M NaCl. 750 µL of ice-cold absolute ethanol was added to precipitate DNA and the samples were centrifuged for 5 min. at 13,000 rpm at + 4 °C. The pellets were washed twice with 750 µL of 70% ethanol and centrifuged under the above conditions to remove residual salt. The tubes were incubated at 55 °C for approximately 2 h with the caps open to allow the excess ethanol to evaporate. In the final step of isolation, DNA pellets were suspended in 50 µL of TE buffer (10 mM Tris–HCl, 1 mM EDTA, pH 7.6) and stored at − 20 °C until analysis.

The DNA solution was prepared by gentle shaking in 0.01 M sodium nitrate solution. DNA-cypermethrin interaction was evaluated by investigating the change in absorbance of mixtures containing DNA and different concentrations of cypermethrin (1:1, 1:2, 1:4). UV absorption spectrum of DNA-cypermethrin complex in the range of 220–300 nm was obtained^30^. UV absorption spectra were recorded on the Mapada UV-6100PCS double beam spectrophotometers.

### Statistical analysis

Statistical analysis of the data obtained as a result of the experimental procedures was carried out using the SPSS for Windows V 22.0 (SPSS Inc, Chicago, IL, USA) package program. One-way ANOVA and Duncan tests were used to evaluate the statistical differences between the experimental groups, respectively. Obtained data were shown as mean ± SEM and were considered statistically significant when *p* values were < 0.05.

## Results and discussion

### Physiological parameters

The effects of cypermethrin on selected physiological parameters in Swiss albino mice are shown in Table 1. The highest body weight gain (+ 9.30 g), liver weight gain (+ 2.16 g) and kidney weight gain (+ 1.25 g) were measured in the control group. In the cypermethrin applied groups, a decrease was observed in the physiological parameters depending on the cypermethrin dose. Compared to the control group, the greatest reduction in body and organ weights among the cypermethrin-administered groups was observed in Group IV, which was treated with 250 mg/kg b.w of cypermethrin. Compared to the control group, body weight decreased 4.3 times (77%), liver organ weight 1.7 times (41%) and kidney organ weight 1.9 times (46%) in Group IV. It was also determined that these decreases were statistically significant (*p* < 0.05). In the literature, some studies are reporting that cypermethrin causes a decrease in body weight and an increase or decrease in organ weights. For example, Sangha et al^.^^7^ reported that in rats exposed to cypermethrin at 50 mg/kg b.w for 2 to 4 weeks, cypermethrin had no effect on the feed and water intake of the rats compared to the control group, but caused significant changes in body and various organ weights. Grewal et al.^6^ investigated the digestive, behavioral, morphological and histopathological effects of male and female albino rats (*Rattus norvegicus*) exposed to cypermethrin at doses of 5 and 20 mg/kg/day orally for 30 days. They reported that cypermethrin caused varying rates of mild to moderate toxic symptoms and behavioral changes in male and female rats, low dose of cypermethrin induced very mild toxicosis characterized by intermittent diarrhea, reduced feed intake and heavy eye discharge while high cypermethrin dose caused mild to moderate toxicosis with diarrhea, decreased feed intake, body weight loss, dyspnea, ataxia, eye discharge and salivation. Hussain et al.^31^ reported that body weight decreased considerably in male rats administered cypermethrin at a dose of 500 mg/kg. The results related to body weight obtained from our study are in line with the results of other studies. In other words, the application of cypermethrin caused a decrease in the live weights of the mice depending on the dose.

**Table 1:** Effects of cypermethrin on selected physiological parameters (g).

Parameters	Group I	Group II	Group III	Group IV
Initial body weight	32.48 ± 0.72	32.65 ± 0.73	32.96 ± 0.71	31.98 ± 0.71
Final body weight	41.78 ± 0.81	39.77 ± 0.80	37.61 ± 0.78	34.14 ± 0.77
Body weight gain	+ 9.30^a^	+ 7.12^b^	+ 4.65^c^	+ 2.16^d^
Liver weight	2.16 ± 0.10^a^	1.85 ± 0.09^b^	1.52 ± 0.06^c^	1.27 ± 0.06^d^
Kidney weight	1.25 ± 0.09^a^	1.10 ± 0.07^b^	0.86 ± 0.06^c^	0.67 ± 0.05^d^
F.C. 7th day (g)	147.00	142.50	137.00	132.60
F.C. 14th day (g)	151.20	136.40	130.10	125.70
F.C. 21st day (g)	155.40	130.90	124.70	116.00
F.C. 28th day (g)	161.70	127.60	119.00	107.80

*Group I: control, Group II: 62.5 mg/kg b.w cypermethrin, Group III: 125 mg/kg b.w cypermethrin, Group IV: 250 mg/kg b.w cypermethrin. F.C: feed consumption. For each group, 210 g of feed was put into the cages weekly. Values are shown as mean ± SEM (n = 6). Data were analyzed with SPSS computer program using Duncan test and ANOVA variance analysis. The averages shown with different letters^(a–d)^ on the same line are statistically significant (*p* < 0.05).

**Figure 2:**
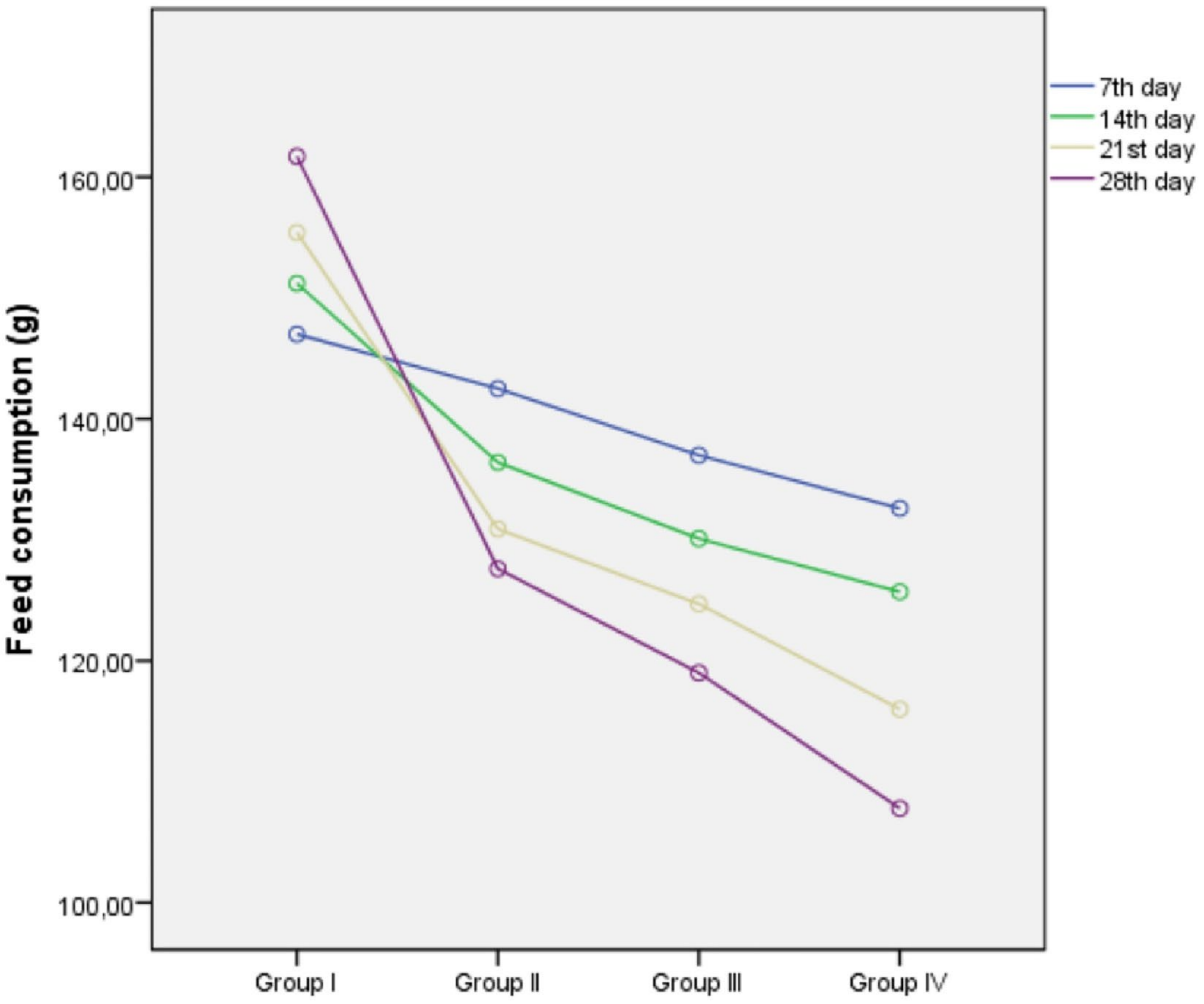
The effects of cypermethrin on weekly feed consumption of mice (g). *Group I: control, Group II: 62.5 mg/kg b.w cypermethrin, Group III: 125 mg/kg b.w cypermethrin, Group IV: 250 mg/kg b.w cypermethrin.

The decrease observed in body weight is thought to be due to the decrease in feed intake of mice in the cypermethrin treated groups. Because in the daily observations, it was determined that the feed consumption of the mice in Group IV, which was administered cypermethrin at a dose of 250 mg/kg b.w, on the 28th day decreased by about 1/3 compared to the control group (Table 1 and Fig. 2). Our results regarding organ weights differ from the results of other studies in that cypermethrin caused a decrease in both liver and kidney weights of mice. As is known, the liver and kidneys are the main detoxification organs. The process of removing toxic agents from the body naturally takes place through these organs. It is thought that the main reason for the decrease in liver and kidney organ weights in mice treated with cypermethrin is the excessive work of these organs to remove cypermethrin from the body or the damage caused by cypermethrin in these organs.

### Serum parameters and cell viability test

The effect of cypermethrin on selected biochemical parameters is shown in Table 2. AST and ALT enzyme activities, BUN and creatinine levels increased significantly compared to the control group, depending on the increase in the doses of cypermethrin administered. It was also determined that these increases were statistically significant (*p* < 0.05). The highest increase in all four measured biochemical parameters was detected in Group IV, which was exposed to 250 mg/kg b.w of cypermethrin. When compared to the control group, approximately 44% increase in AST enzyme activity, approximately 56% increase in ALT enzyme activity, approximately 106% increase in BUN level and approximately 276% increase in creatinine level were observed in Group IV. There are some studies in the literature that confirm these results that we have obtained. Amir et al.^32^ have reported that serum AST and ALT activities were increased in Wistar rats fed 0.00 mg/kg (negative control), 0.05 mg/kg, 0.60 mg/kg, 1.10 mg/kg, 1.60 mg/kg and 2.15 mg/kg doses of cypermethrin and 1.73 mg (positive control) of cypermethrin-containing fish meat, and that these increases were highest at 2.15 mg/kg dose of cypermethrin. Abdou et al.^33^ reported that serum AST and ALT enzyme activities, BUN and creatinine levels increased considerably in rats exposed to cypermethrin at a dose of 12 mg/kg by oral gavage for 30 consecutive days. Abdul-Hamid et al.^34^ found statistically significant increases in serum AST, ALT and ALP enzyme activities in male albino rats weighing approximately 120–150 g exposed to cypermethrin at 30 mg/kg dose for 28 consecutive days.

**Table 2:** Effects of cypermethrin on selected biochemical and oxidative stress parameters.

Parameters	Group I	Group II	Group III	Group IV
AST (U/L)	75.00 ± 1.98^d^	83.00 ± 2.01^c^	94.00 ± 2.44^b^	108.00 ± 3.03^a^
ALT (U/L)	50.00 ± 1.32^d^	58.00 ± 1.50^c^	65.00 ± 1.70^b^	78.00 ± 2.09^a^
BUN (mg/L)	120.00 ± 3.19^d^	150.00 ± 3.88^c^	185.00 ± 4.27^b^	247.00 ± 5.14^a^
Creatinine (mg/L)	4.25 ± 0.52^d^	7.56 ± 0.63^c^	10.85 ± 0.80^b^	16.00 ± 1.03^a^

*Group I: control, Group II: 62.5 mg/kg b.w cypermethrin, Group III: 125 mg/kg b.w cypermethrin, Group IV: 250 mg/kg b.w cypermethrin. Values are shown as mean ± SEM (n = 6). Data were analyzed with SPSS computer program using Duncan test and ANOVA variance analysis. The averages shown with different letters^(a–d)^ on the same line are statistically significant (*p* < 0.05).

AST and ALT are two indicator enzymes used as the most important indicator of liver damage. In case of any disease, drug use, chemical or radioactive damage in the liver, the activity of these two enzymes in the blood increases. BUN and creatinine are used as markers of kidney damage. Ammonia is formed as a result of the breakdown of proteins during digestion. Ammonia, which is extremely toxic to the body and contains nitrogen, reaches the liver through the blood, and the liver converts ammonia to urea, which is less toxic to the body. The urea reaching the kidneys through the blood is filtered here and excreted out of the body through the urine. Creatinine is a substance that occurs in muscle cells and is excreted from the body through the kidneys. Damage to the kidneys causes an increase in blood urea and creatinine levels. In this study, the increase in AST and ALT enzyme activities, and BUN and creatinine levels in the serum of mice administered cypermethrin are indicators of destruction or damage in liver and kidney cells.

**Figure 3:**
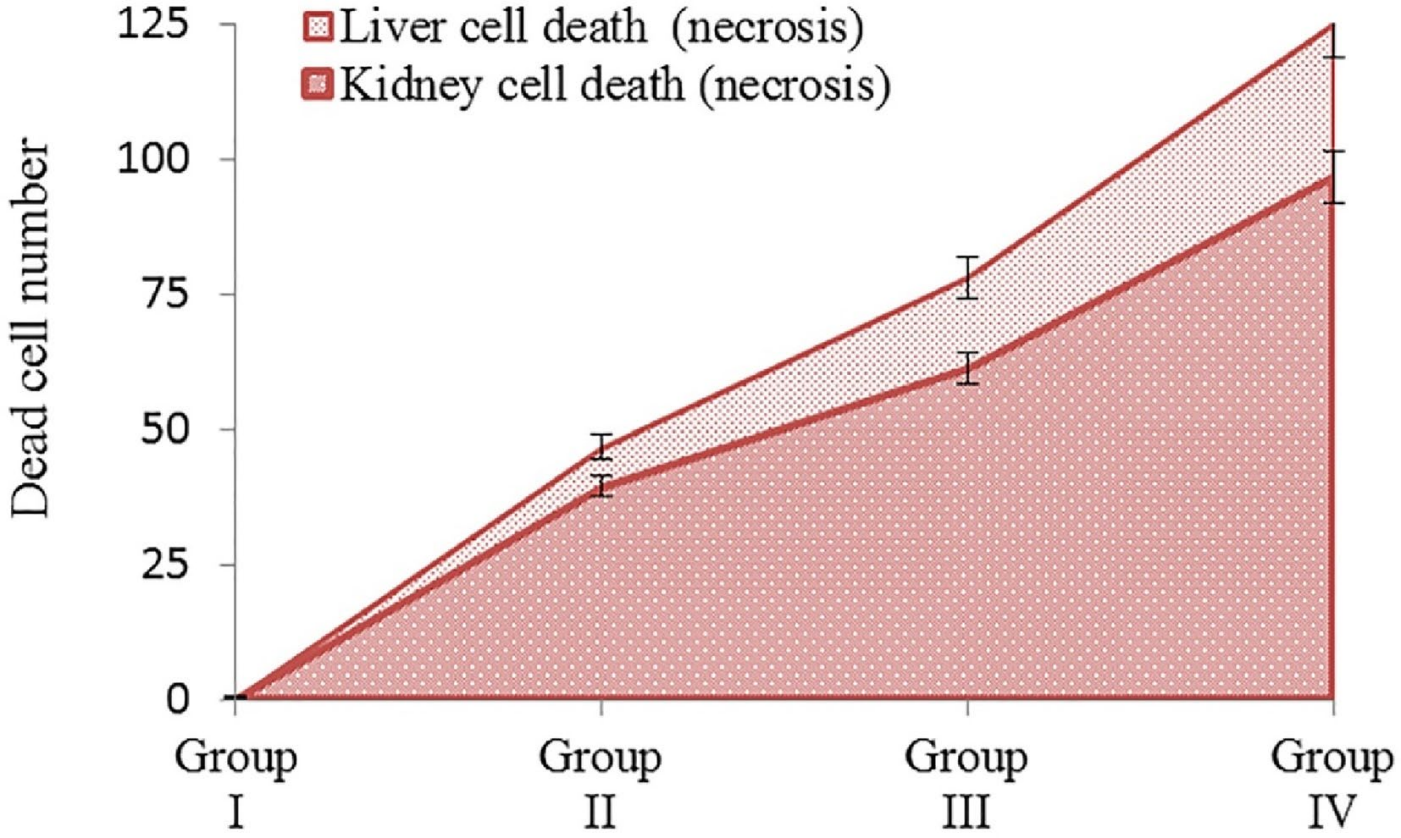
Cell death induced by cypermethrin in liver and kidney cells. *Group I: control, Group II: 62.5 mg/kg b.w cypermethrin, Group III: 125 mg/kg b.w cypermethrin, Group IV: 250 mg/kg b.w cypermethrin. 100 cells were counted for each mouse in each group, a total of 600 cells. Values are shown as mean ± SEM (n = 6).

In this study, TBT test was performed to identify potential cell damage (cell viability) induced by cypermethrin in liver and kidney tissues. In the TBT test, a suspension of cells is mixed with the dye and then it is determined whether the cells have taken up the dye. If there is cell death, then the dye enters the cell through the damaged membrane, causing staining of the cell. In living cells, however, it does not allow staining as it cannot enter the cell. When examined with a light microscope, only the membranes of living cells are observed in blue color, while dead cells are observed completely in blue color. In this study, no dead (necrotic) cells were found in the liver and kidney cells of the control group (group I) mice. However, 125 dead (necrotic) cells were observed in liver cells and 96.7 in kidney cells of Group IV mice exposed to 250 mg/kg dose of cypermethrin (Fig. 3). Our results are supported by the results of histopathological studies carried out by other researchers on the effects of cypermethrin on liver and kidney tissues. Grewal et al.^6^ observed hepatic lamina irregularity, increase in sinusoids, hemorrhage, shrinkage of glomeruli, sagging of renal convoluted tubules, and necrosis in liver and kidney tissues of male and female albino rats exposed to cypermethrin at 5 and 20 mg/kg/day doses orally for 30 days. Abdul-Hamid et al.^34^ determined that the application of cypermethrin promoted extensive vacuolar degeneration of hepatocytes, fat exchange, blood vessel occlusion and fibrosis in the livers of rats. They also observed that it caused a decrease in the amount of glycogen, protein and DNA, mitochondria damage and nuclear changes. Abdou et al.^33^ reported that cypermethrin treatment causes histological changes by promoting liver and kidney damage.

### Antioxidant/oxidant dynamics

In order to determine the effect of cypermethrin on antioxidant and oxidant balance in kidney and liver tissues, MDA and GSH levels were measured in tissues. The effects of cypermethrin on antioxidant/oxidant dynamics are shown in Table 3. Cypermethrin exposure caused statistically significant (*p* < 0.05) increases in MDA levels, which is the main indicator of lipid peroxidation in liver and kidney tissues. It has been determined that these increases are directly proportional to the applied cypermethrin dose. Compared to the control group, the MDA level in liver tissues increased approximately 1.17 times in Group II, 1.37 times in Group III and approximately 1.63 times in Group IV. Similarly, in kidney tissues, it increased approximately 1.11 times in Group II, 1.29 times in Group III and 1.41 times in Group IV. It was also determined that the increases in MDA levels were higher in liver tissues than in kidney tissues.

**Table 3:** Effects of cypermethrin on selected biochemical and oxidative stress parameters.

Parameters	Group I	Group II	Group III	Group IV
MDA_Liver_ (nmol/g)	0.215 ± 0.04^d^	0.252 ± 0.07^c^	0.295 ± 0.10^b^	0.351 ± 0.15^a^
MDA_Kidney_ (nmol/g)	0.175 ± 0.02^d^	0.194 ± 0.03^c^	0.226 ± 0.06^b^	0.247 ± 0.11^a^
GSH_Liver_ (mg/g)	0.275 ± 0.23^a^	0.235 ± 0.17^b^	0.210 ± 0.14^c^	0.168 ± 0.10^d^
GSH_Kidney_ (mg/g)	0.246 ± 0.24^a^	0.218 ± 0.15^b^	0.195 ± 0.10^c^	0.162 ± 0.05^d^

*Group I: control, Group II: 62.5 mg/kg b.w cypermethrin, Group III: 125 mg/kg b.w cypermethrin, Group IV: 250 mg/kg b.w cypermethrin. MDA: malondialdehyde, GSH: glutathione. Values are shown as mean ± SEM (n = 6). Data were analyzed with SPSS computer program using Duncan test and ANOVA variance analysis. The averages shown with different letters^(a–d)^ on the same line are statistically significant (*p* < 0.05).

Cypermethrin exposure caused statistically significant (*p* < 0.05) decreases in GSH levels, which is an important indicator of damage caused by free radicals and reactive oxygen species in the cell. With the increase in the dose of cypermethrin administered, GSH levels decreased considerably compared to the control group. The level of GSH in liver tissues decreased approximately 1.17 times in Group II, approximately 1.31 times in Group III, and approximately 1.64 times in Group IV compared to the control group. Similarly, in kidney tissues, it decreased approximately 1.13 times in Group II, 1.26 times in Group III and 1.52 times in Group IV. It was also determined that the decreases in GSH levels were higher in liver tissues than in kidney tissues. Our results regarding the change in the levels of selected oxidative stress parameters by cypermethrin are in agreement with the results of similar studies. Abdou et al.^34^ reported a decrease in GSH level and antioxidant enzyme activities in rats exposed to 12 mg/kg dose of cypermethrin by oral gavage for 30 days. Abdul-Hamid et al.^34^ found an increase in MDA levels and a decrease in antioxidant enzymes SOD and GPx activity in male albino rats exposed to cypermethrin at a dose of 30 mg/kg for 28 days. Ateşşahin et al.^35^ determined that rats exposed to 50 mg/kg dose of cypermethrin during 5 consecutive days had significant increases in MDA levels in liver, kidney and brain tissues, and in GSH-Px activity in liver tissue and erythrocyte cells. GSH is a tripeptide produced in liver cells. It is a powerful antioxidant responsible for the detoxification process that takes place in the liver and GSH is known as the main anti-oxidant^36,37^. MDA is the end product of lipid peroxidation. Lipid peroxidation is a process of oxidative breakdown of membrane phospholipids. Free radicals break the double bonds in unsaturated fatty acids in the presence of oxygen and MDA, which is a by-product of unsaturated fatty acid oxidation, is formed. MDA cross-links proteins, enzymes and phospholipids, causing membrane polymerization, disruption of ion transport and enzyme activity. As a result, the structure of the cell membrane deteriorates and its permeability is adversely affected^38,39^. On the other hand, it has been reported that many chemicals and radioactive agents cause an increase in MDA levels and a decrease in GSH levels in cells by generating free radicals and reactive oxygen species (ROS). In this study, it is thought that the increase in MDA levels and decreases in GSH levels measured in liver and kidney tissues as a result of cypermethrin exposure is due to the potential of cypermethrin to form free radicals and ROS. In some studies, it has been reported that cypermethrin shows its toxic effects through the formation of ROS^40^.

### Cytotoxic and genotoxic effects

**Figure 4:**
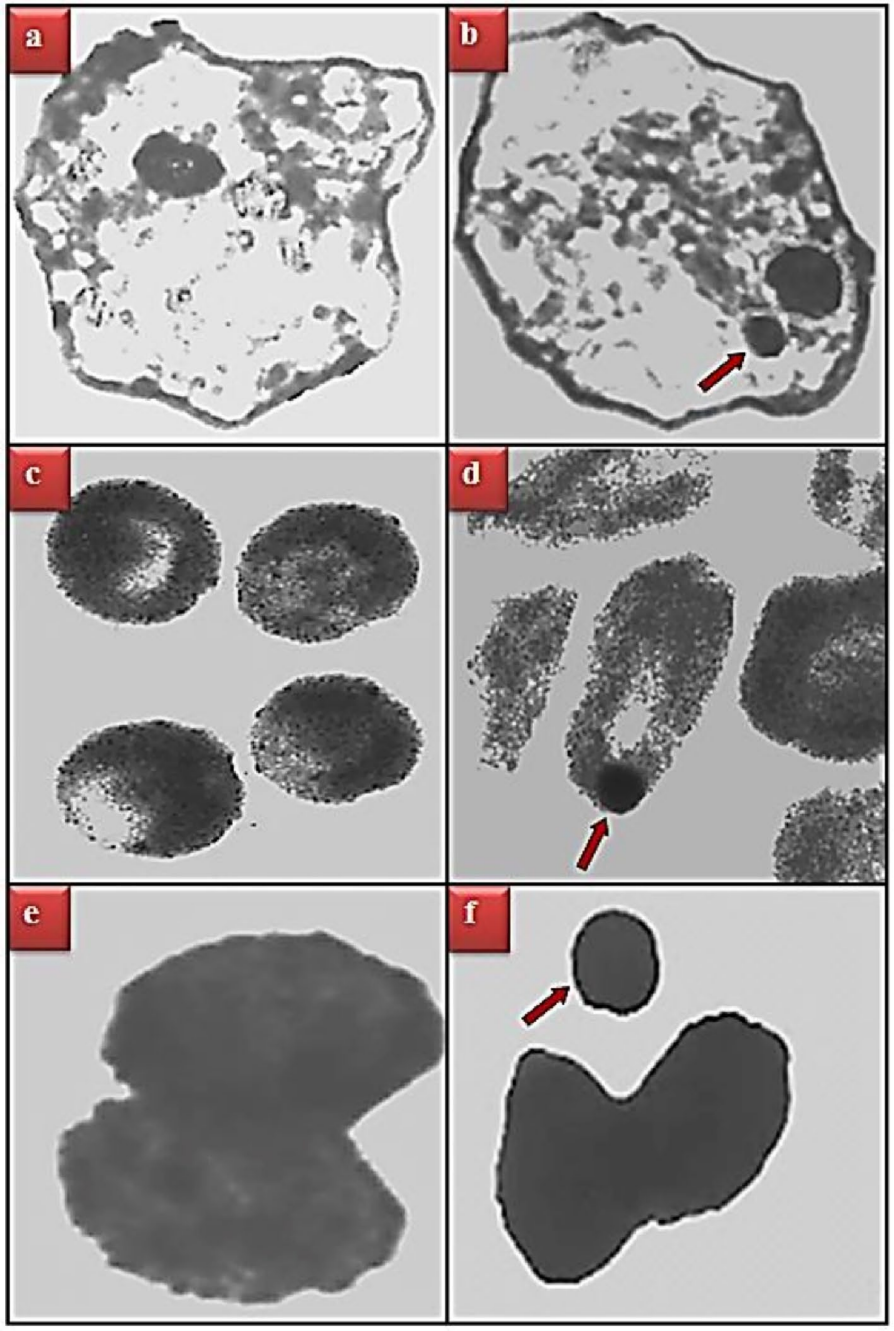
MN formation promoted by cypermethrin. Buccal mucosal epithelium normal appearance (**a**), buccal mucosal epithelium with MN (**b**), erythrocyte cell normal appearance (**c**), erythrocyte cell with MN (**d**), leukocyte cell normal appearance-basophil (**e**), leukocyte cell-basophil with MN (**f**).

The genotoxic effects of cypermethrin are shown in Table 4 and Fig. 4. Cypermethrin exposure induced MN formation in buccal mucosa epithelium, erythrocyte and leukocyte cells and CAs in bone marrow cells depending on the dose. The highest MN formation promoted by cypermethrin was observed in leukocyte > erythrocyte > buccal mucosa epithelial cells, respectively. The main reason why MN formation is observed at least in buccal mucosal epithelial cells is that the mice do not keep cypermethrin in their mouths too much when they take it orally, they swallow it immediately, and therefore the epithelial cell chromosomes do not have the opportunity to interact with cypermethrin too much. In addition, considering that erythrocyte cells also lose their nuclei during their transition from the bone marrow where they are produced to the blood, it can be explained as the main reason why less MN formation is observed compared to leukocyte cells. In all three cell types, the number of MNs increased considerably compared to the control group, and these increases were found to be statistically significant (*p* < 0.05). The greatest increase in MN count in all three cell types was observed in Group IV exposed to 250 mg/kg b.w dose of cypermethrin. MN was counted at the rate of 20.52 ± 0.96 in buccal mucosal epithelium cells, 53.96 ± 2.23 in erythrocyte cells and 76.34 ± 2.81 in leukocyte cells of Group IV, respectively. As a result of the examination of chromosomes obtained from bone marrow cells under the light microscope, it was determined that cypermethrin encourages chromosomal damages in the form of break, fragment, ring chromosome, gap, acentric and dicentric. The greatest effect of cypermethrin on CAs occurred in the form of fragment formation. While no break formation was observed in the control group, 16.20 ± 0.91, 31.98 ± 1.45 and 48.62 ± 1.94 fragments were observed in the groups exposed to 62.5, 125 and 250 kg/mg b.w doses of cypermethrin, respectively. In addition, it was determined that the number of CAs caused by cypermethrin increased depending on the application dose and these increases were statistically significant (*p* < 0.05). There are some studies in the literature that confirm our results. Chauhan et al.^41^ investigated the genotoxic effects of 22, 44 and 67 mg/kg doses of cypermethrin and Quinalphos mixture in male albino mice. As a result, they reported that cypermethrin administration dose-dependently inhibited the MI, especially promoted CAs such as chromatid break and fragment, and caused MN formation in polychromatic erythrocyte cells. Amer et al.^42^ investigated CAs and sister chromatid exchanges induced by cypermethrin in mouse spleen and bone marrow cells and in vitro in cultured mouse spleen cells. As a result, CAs in the form of chromatid and chromosome gaps, fragments and tetraploidy were observed in spleen and bone marrow cells. They also found that there was a significant dose-dependent increase in the frequency of sister chromatid exchanges (SCE) in mouse bone marrow cells. Similar results were also found in mouse spleen cells in cell cultures. Giri et al.^43^ reported that 5, 10 and 20 mg/kg doses of cypermethrin in the in vivo mouse test system caused significant increases in the frequency of SCE in bone marrow cells depending on the dose.

**Table 4:** Genotoxic effects of cypermethrin.

Parameters	Group I	Group II	Group III	Group IV
Buccal epithelium MN	0.00 ± 0.00^d^	7.54 ± 0.60^c^	11.28 ± 0.80^b^	20.52 ± 0.96^a^
Erythrocyte MN	0.00 ± 0.00^d^	16.76 ± 0.87^c^	32.50 ± 1.46^b^	53.96 ± 2.23^a^
Leukocyte MN	0.34 ± 0.47^d^	24.28 ± 1.03^c^	40.38 ± 1.89^b^	76.34 ± 2.81^a^
Break	0.00 ± 0.00^d^	16.20 ± 0.91^c^	31.98 ± 1.45^b^	48.62 ± 1.94^a^
Fragment	0.00 ± 0.00^d^	10.84 ± 0.79^c^	21.46 ± 0.96^b^	35.22 ± 1.62^a^
Ring	0.00 ± 0.00^d^	6.15 ± 0.61^c^	13.75 ± 0.87^b^	24.76 ± 1.45^a^
Gap	0.00 ± 0.00^d^	3.92 ± 0.47^c^	7.40 ± 0.56^b^	15.35 ± 0.96^a^
Acentric	0.00 ± 0.00^d^	1.16 ± 0.46^c^	4.38 ± 0.65^b^	10.84 ± 1.05^a^
Dicentric	0.00 ± 0.00^d^	0.34 ± 0.39^c^	3.16 ± 0.58^b^	7.62 ± 0.79^a^

*Group I: control, Group II: 62.5 mg/kg b.w cypermethrin, Group III: 125 mg/kg b.w cypermethrin, Group IV: 250 mg/kg b.w cypermethrin. MN: Micronucleus. 1000 cells were analyzed for MN frequency and 600 cells were analyzed for CAs in each group. Values are shown as mean ± SEM (n = 6). Data were analyzed with SPSS computer program using Duncan test and ANOVA variance analysis. The averages shown with different letters^(a–d)^ on the same line are statistically significant (*p* < 0.05).

**Figure 5:**
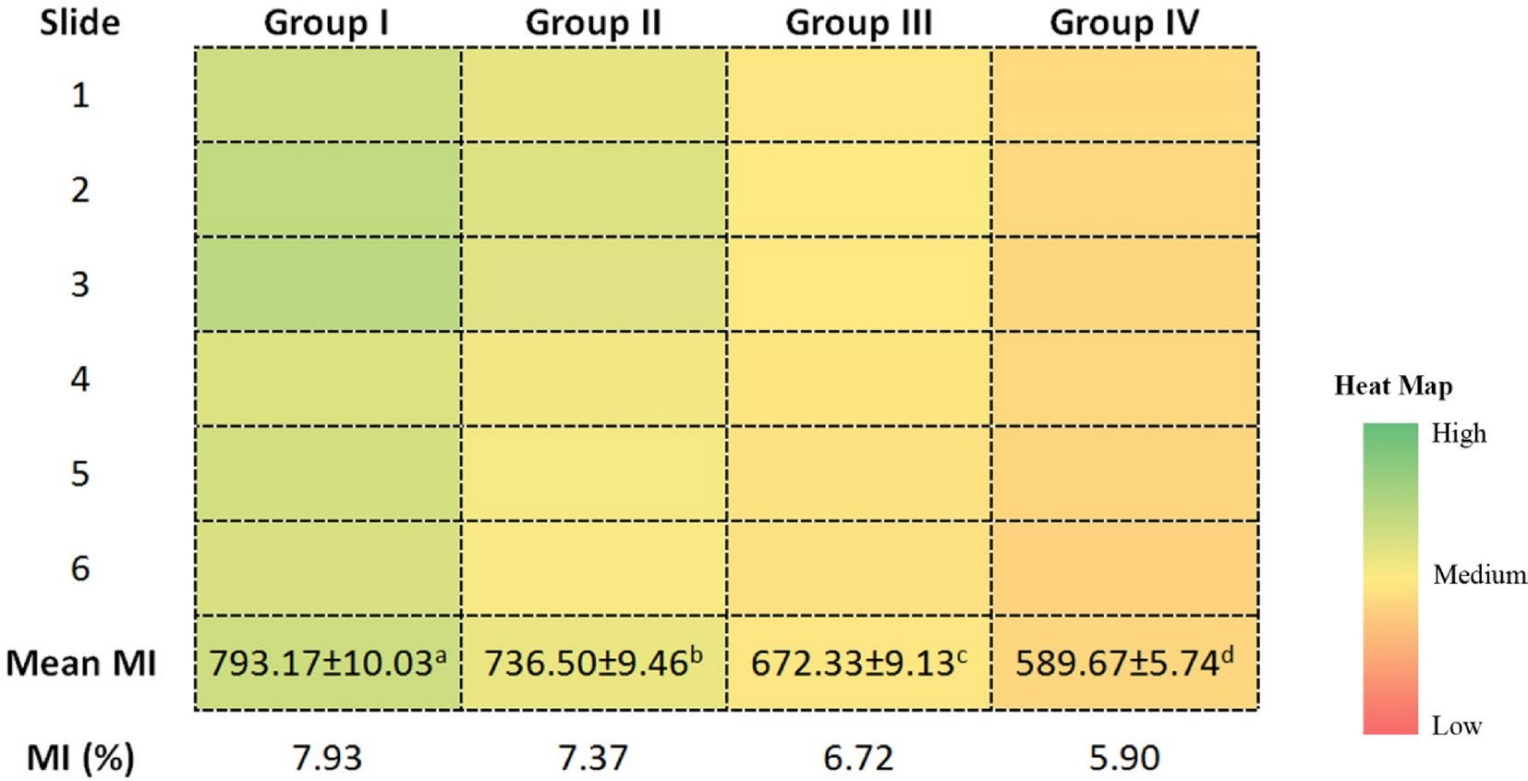
The effects of cypermethrin on MI. *Group I: control, Group II: 62.5 mg/kg b.w cypermethrin, Group III: 125 mg/kg b.w cypermethrin, Group IV: 250 mg/kg b.w cypermethrin. MI was calculated by analyzing 1000 cells per animal (for a total 6000 cells per group). Values are shown as mean ± SEM (n = 6). Data were analyzed with SPSS computer program using Duncan test and ANOVA variance analysis.

The heat map showing the effect of cypermethrin on MI, an indicator of cell proliferation, is given in Fig. 5. Approximately 793 cells out of 6000 cells counted in the control group were at the stage of division, and this number decreased in a dose-dependent manner with cypermethrin administration. In the groups administered with 62.5, 125 and 250 kg/mg b.w doses of cypermethrin, the MI was determined as 7.37%, 6.72% and 5.90%, respectively. The most toxic effect in terms of cell proliferation was observed in Group IV. The MI-reducing effect of cypermethrin can be explained by different mechanisms. The most important of these mechanisms is the interruptions in the cell cycle as a result of MN and CAs formations induced by cypermethrin, which was demonstrated by the previous analyzes in this study.

### DNA fragmentation

**Figure 6:**
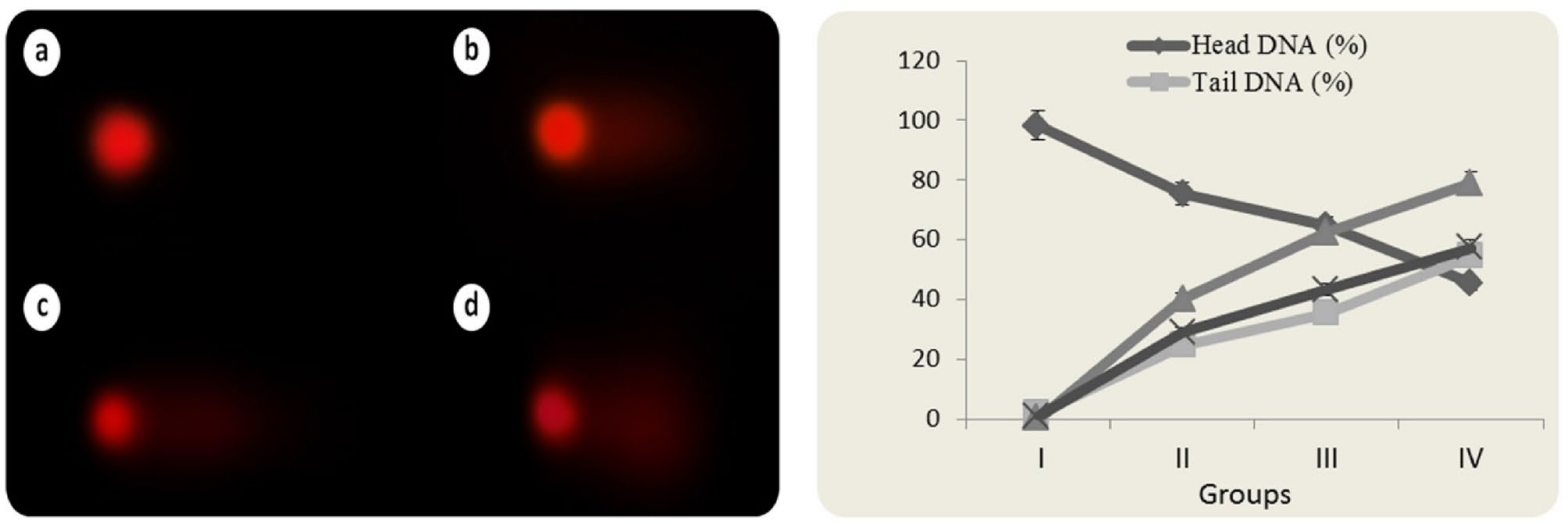
Comet assay analysis in leukocyte cell nucleus of cypermethrin-treated mice (**a** control, **b** 62.5 mg/kg b.w cypermethrin, **c** 125 mg/kg b.w cypermethrin, **d** 250 mg/kg b.w cypermethrin).

Cypermethrin induced DNA fragmentation was investigated with Comet assay. The percentage of tail DNA, tail moment, and olive tail moment were used to assess the DNA damage in the leukocyte cell nucleus of Swiss albino mice. In the comet test, DNA strand breaks are observed as damaged DNA migrates at a different rate from undamaged DNA during electrophoresis. When damaged DNA is subjected to electrophoresis, the damaged DNA moves away from the undamaged DNA containing the nucleoid body, resembling a "comet" structure. In the comet structure, the undamaged DNA nucleoid portion is called the "head" and the following damaged DNA line is called the "tail"^44^. The percentage of DNA in the tail is directly proportional to the percentage of DNA damage that has occurred in a particular cell. The comet assay data of cypermethrin treated groups are given in Fig. 6. In the control group (Group I), the average percentage of tail DNA was calculated as 1.74. As can be shown, exposure to cypermethrin induced DNA damage in a dose-dependent manner at all doses. The percentage of tail DNA, tail moment and olive tail moment increased dramatically in leukocyte cells of mice treated with cypermethrin at dose of 62.50 mg/kg b.w and reached a mean of 24.68, 40.36 and 28.94, respectively. The tail DNA percentage increased with greater cypermethrin exposure levels, reaching 35.42 in Group III and 54.62 in Group IV. Cypermethrin dose increases in Groups III and IV resulted in massive increases in the tail moment and olive tail moment parameters. All these comet assay data showed that cypermethrin administration caused DNA fragmentation in leukocyte cell nuclei and this was dose dependent. Differences in tail DNA percentage, tail moment and olive tail moment parameters between groups are statistically significant (*p* < 0.05).

**Figure 7:**
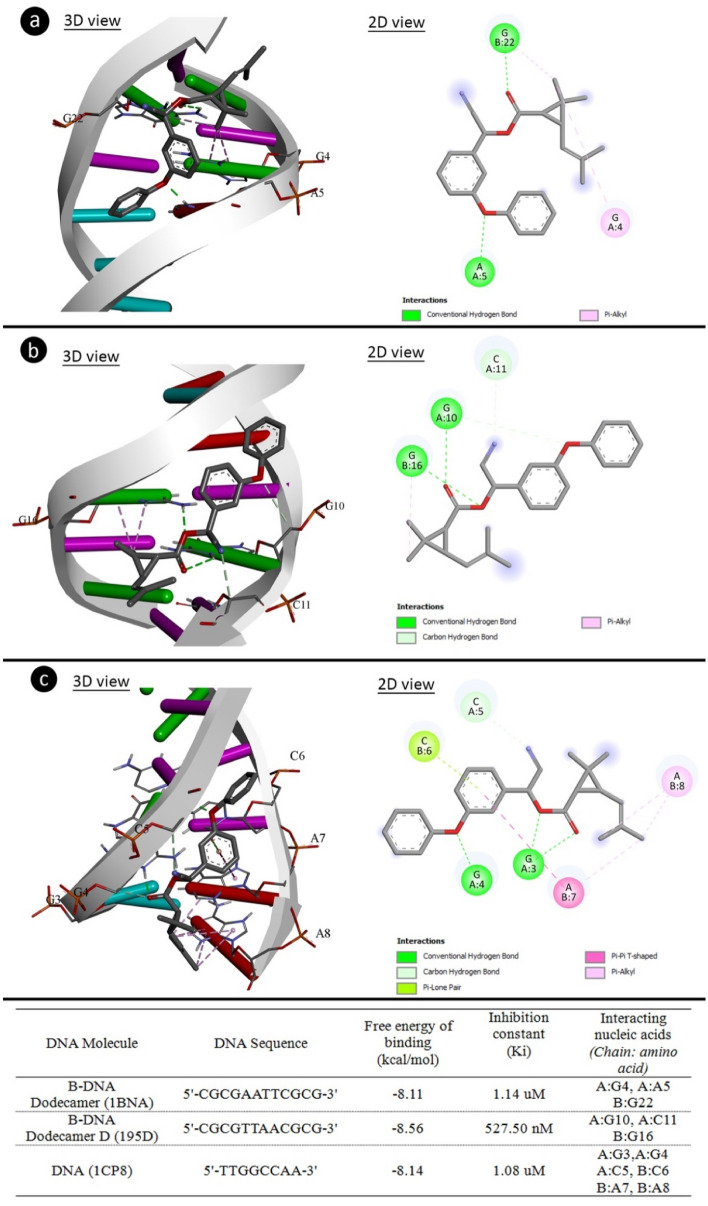
The molecular interactions of cypermethrin with DNA molecules. 1BNA (**a**), 195D (**b**), 1CP8 (**c**).

As a result of the comet and CAs analysis, it was determined that the application of cypermethrin caused abnormalities in the structure of DNA and chromosomes. To elucidate this toxic effect of cypermethrin, molecular docking studies of cypermethrin with DNA were performed. The binding affinities of cypermethrin on the DNA structure were investigated in order to rationalize the genotoxic mechanism of cypermethrin at the molecular level. Figure 7 shows the evidence of potential interactions of cypermethrin with DNA molecules. Cypermethrin had contact with B-DNA dodecamer (1BNA) with -8.11 kcal/mol binding energy and inhibition constant of 1.14 uM. Cypermethrin exhibited hydrogen bond interactions with A5 base in the chain A and G22 in chain B, as well as hydrophobic interactions with G4 in chain A and G22 in chain B. Cypermethrin was bound hydrogen bonding interactions to bases G4 and C11 (chain A) and hydrophobic interactions to G16 base (chain B) of B-DNA Dodecamer D (195D) with binding energy − 8.56 kcal/mol and inhibiting constant of 527.50 nM. In DNA (1CP8), cypermethrin interaction has occurred with -8.14 kcal/mol binding energy and inhibition constant of 1.08 uM, G3, G4 and C5 bases (chain A) formed hydrogen bond interactions, C6 (chain B) base Pi-Lone Pairbond and A7 and A8 bases formed hydrophobic interactions. It also showed that cypermethrin can affect the structure of DNA by binding to regions rich in G-A, G-C, G-G-C and C-A-A bases. The findings of molecular docking studies with cypermethrin and various DNA molecules demonstrated that cypermethrin can interact with both the same and distinct strands in DNA molecules, as well as the potential for intercalation. Intercalation occurs by stacking chemicals between base pairs in DNA molecules without forming any covalent bonds between the DNA and chemical agent^45^. Such DNA intercalators are not DNA adductors, but the intercalation of chemicals may be promutagenic. Intercalation also causes the supercoiled DNA to unravel, which can ultimately prevent DNA from being recognized by DNA-binding proteins and other regulatory factors. Intercalator agents such as cypermethrin have diverse and multiple biological effects on DNA. Inhibition in DNA and RNA synthesis, protein-associated DNA breaks and frameshift mutations are some of these effects^45,46^. Especially DNA breaks lead to high CAs and MN formations.

**Figure 8:**
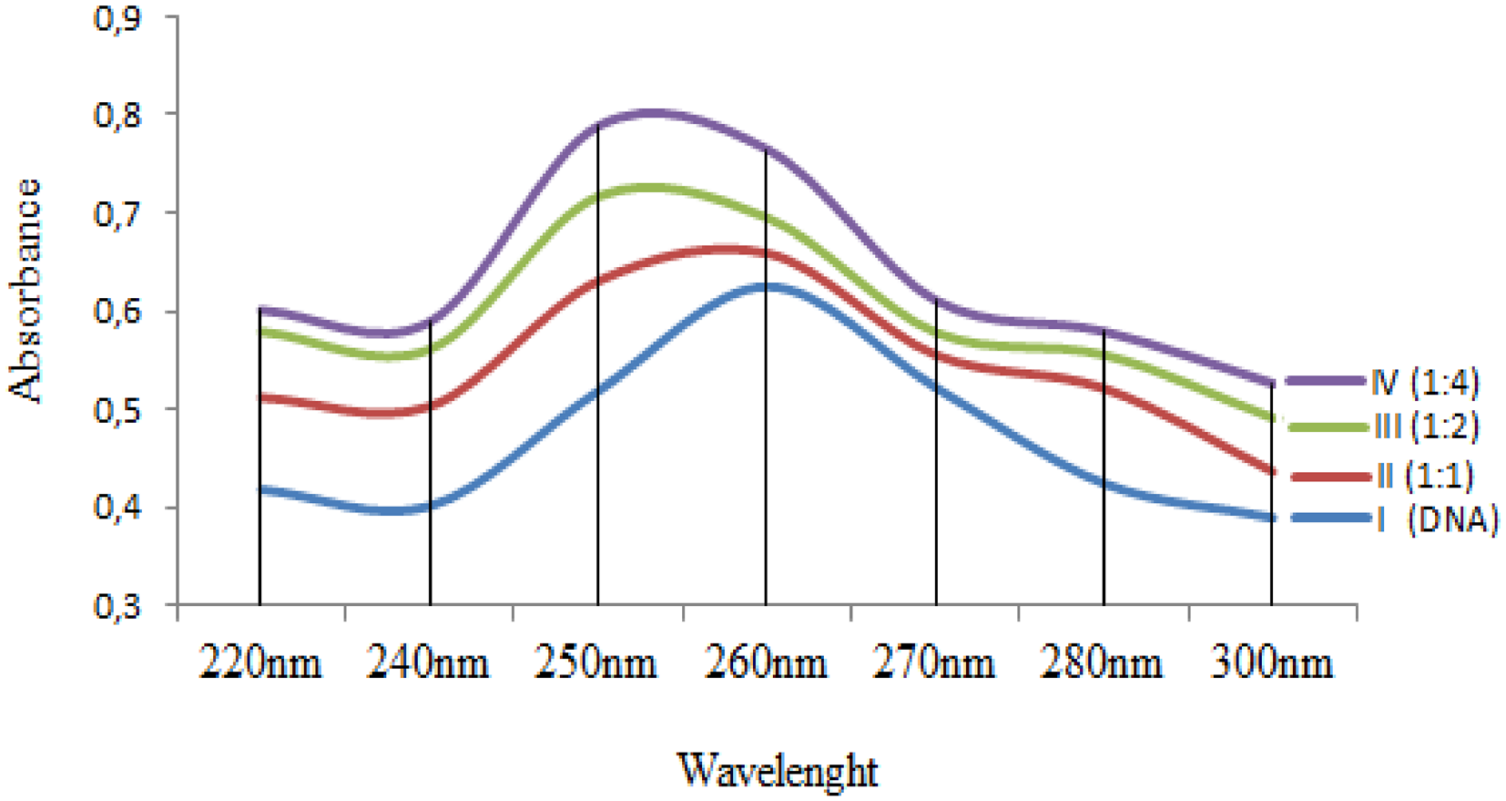
UV absorption spectrum of cypermethrin and DNA interaction.

The interaction of cypermethrin and DNA, shown by molecular docking, was also supported by the UV absorption spectrum and the results are given in Fig. 8. Addition of cypermethrin to the DNA solution caused bathochromic and hyperchromic shifts in the UV spectrum. The bathochromic shift was from 260 to 250 nm, and the hyperchromic shift was from 0.625 to 0.789. As the cypermethrin ratio increased, the shift intensity also increased. The bathochromic shift confirms the cypermethrin-DNA interaction. Spectral hyperchromicity of DNA (increase in A_260_) also indicates partial instability of the secondary structure of DNA resulting from the interaction^47,48^. Binding of cypermethrin to DNA probably occurs as a result of the strong polarization associated with the chlorine atoms. This polarization can cause destabilization of DNA structure, disruption of DNA integrity and uncoiling of the DNA helix, resulting in chromosomal damages. In similar studies, it has been reported that cypermethrin interacts with the calf thymus DNA and there are shifts in the UV spectra after this interaction^30^.

## Conclusion

In conclusion, cypermethrin induced toxicity in Swiss albino mice by causing statistically significant changes in selected physiological, biochemical and cytogenetic parameters depending on the dose. The data obtained as a result of the experimental procedures are also compatible with the results of similar studies. However, similar studies of cypermethrin toxicity have focused on only one parameter, namely the physiological or biochemical or genotoxic effects of cypermethrin. This study is the most comprehensive study that deals with the physiological, biochemical and genotoxic effects of cypermethrin as a whole. In addition, MN formation was investigated especially in polychromatic erythrocyte in the other studies conducted to determine the genotoxic effects of cypermethrin. The detection of MN formation by cypermethrin, especially in the buccal mucosal epithelium and blood leukocyte cells is the first in the literature. All parameters tested within the scope of the study were correlated with each other and the toxicity mechanism was evaluated. With molecular docking studies, the mechanism of genotoxic effects has been clarified, and it has been determined that cypermethrin acts as an intercalation agent and disrupts genome integrity. On the other hand, while rats are preferred as the animal organism in many studies on cypermethrin toxicity, the number of studies with albino mice is very few. The fact that this study was conducted on Swiss albino mice will provide an important data entry to the literature when evaluated together with the above-mentioned issues. It is very important that studies investigating the in vivo toxic effects and toxicity mechanism of environmental pollutants and compounds that contaminate organisms take place in the literature. Each chemical substance has a potential toxic effect on living things. The important thing is to illuminate the mechanisms by which this toxic effect occurs. Therefore, this study will be a guide for similar studies.

## Data Availability

The datasets used and/or analyzed during the current study are available from the corresponding author on reasonable request.
